# Pharmacokinetics and bioequivalence evaluation of omeprazole and sodium bicarbonate dry suspensions in healthy Chinese volunteers

**DOI:** 10.1038/s41598-022-27286-5

**Published:** 2023-01-20

**Authors:** Rui Zhang, Pengpeng Guo, Jinping Zhou, Peixia Li, Jing Wan, Chunxiao Yang, Jiali Zhou, Yani Liu, Shaojun Shi

**Affiliations:** 1 Present Address: Department of Pharmacy, Union Hospital, Tongji Medical College, Huazhong University of Science and Technology, Wuhan, 430022 People’s Republic of China; 2 Union Jiangnan Hospital, Huazhong University of Science and Technology, Wuhan, 430022 People’s Republic of China

**Keywords:** Clinical genetics, Pharmaceutics, Clinical trial design

## Abstract

Omeprazole and sodium bicarbonate dry suspension are effective treatments for acid-related disorders. This study compared the bioequivalence and safety of the two formulations of omeprazole and sodium bicarbonate powder and assessed how CYP2C19 gene polymorphisms affect pharmacokinetics (PK). A single-center, randomized, single-dose, 2-sequence and 2-period crossover method was performed in forty healthy Chinese subjects. Blood samples were collected after a single dose for PK (AUC_0–∞_, AUC_0–t_, and C_max_) analysis. The concentrations of Omeprazole in human plasma were determined by HPLC–MS/MS. Besides, the gene polymorphisms of CYP2C19 were assessed by Sanger sequencing. The geometric mean ratios (90% confidence interval) [GMR (95% CI)] of Test/Reference preparation for C_max_: 95.2% (88.48%, 102.43%), AUC_0–t_: 97.47% (94.4%, 101.02%), AUC_0–∞_: 97.68% (94.27%, 101.21%) were within the range of 80.00–125.00%. The non-parametric test showed no statistical difference in T_max_ between the two groups (*p* > 0.05). All drugs were well tolerated, no severe adverse reactions occurred, and no significant differences in adverse events between the two drugs. For CYP2C19 gene polymorphisms, the results showed that of 40 subjects, 12 subjects were extensive metabolizers, 24 were intermediate metabolizers, and 4 were poor metabolizers, the frequency of metabolic genotypes were 30%, 60%, and 10%. And the allele distributions for CYP2C19 were *1, *2, and *3 at 60%, 38.75%, and 1.25%. Both the CYP2C19 alleles and metabolic genotypes were consistent with other studies in Chinese. The results of PK parameters showed that different genotypes of CYP2C19 lead to significant differences in t_1/2_, AUC_0–t_, AUC_0–∞_ and C_max_, but no significant differences in T_max_ in each group. At the same time, we confirmed that the PK parameters of the test and reference had no differences between the males and females. This study has shown that the pharmacokinetic parameters of the two formulations are not significantly different, which showed bioequivalence and exemplary safety. CYP2C19 gene polymorphism significantly differed in the PK parameters of omeprazole sodium bicarbonate powder.

## Introduction

Proton pump inhibitors (PPIs) are widely used to treat a variety of acid-related disorders, including gastroesophageal reflux disease (GERD)^1^, peptic ulcer disease (PUD)^2^, Helicobacter pylori (H.pylori) infections^3^, and the prophylaxis of stress- and NSAID-induced PUD^4–6^. Omeprazole has been widely recognized and used as the first generation of new acid inhibitors once discovered^7^. Omeprazole has been combined with antibiotics such as amoxicillin and clarithromycin to eradicate helicobacter pylori^8^. The main metabolizing enzyme of omeprazole is CYP2C19^9^, and the factors affecting the activity of CYP2C19 include age^9^, medications^10^, etc., which may also influence the metabolism of omeprazole, causing changes in area under curve (AUC) and its activity. The abnormality of the CYP2C19 coding gene is the most crucial and researched pharmacogenetic factor affecting the clearance of omeprazole and its efficacy^10,11^. Because of the differences in the CYP2C19 gene polymorphisms, the subjects can be separated into three groups, extensive metabolizers (EM), intermediate metabolizers (IM), and poor metabolizers (PM)^12^. Of the CYP2C19 genetic polymorphisms, many studies found that due to variations in*2(G681A) and *3(G636A), the enzyme activity was reduced^13–15^. The frequency of CYP2C19*2/*3 associated with non-functions in Asians is 13–23%, much higher than that of Caucasians^16^. Due to the lower activity of CYP2C19 and slower drug clearance, the omeprazole exposure of plasma in PM could be higher, leading to differences in efficacy^17,18^. Therefore, it is worthwhile to pay attention to the metabolic genotypes of CYP2C19 in Chinese volunteers, and to observe its correlation with adverse reactions, thereby providing a basis for clinically rational drug use and individualized treatment.

The pharmacological effect of omeprazole is mainly through forming a covalent complex with H+-K+-ATPase in the activated form of sulfonamide derivatives, which irreversibly inactivates the latter and blocks the final step of gastric acid secretion to reach the acid suppression effect^19^. Until now, all available delayed-release PPIs are enteric-coated preparations administered orally because they can be destroyed easily in the stomach, including oral suspensions, disintegrating tablets, and capsules. Different intestinal coverings are necessary to protect unstable PPI from acid degradation in the stomach but have the probable detriment of delaying the absorption of PPI^20^. The FDA approved the American Santarus Company’s Omeprazole Sodium Bicarbonate Dry Suspension for the market in June 2004; the product name is “ZEGERID”, and the indications are gastroesophageal reflux disease, active benign gastric ulcer, etc., which have been confirmed in several studies^21,22^. This new immediate-release suspension of omeprazole is protected from stomach acid degradation by sodium bicarbonate, which increases the pH in the stomach to protect the omeprazole, facilitating its rapid absorption and onset of antisecretory effect^21^.

This study aimed to access the pharmacokinetics bioequivalence and safety of the omeprazole sodium bicarbonate dry suspension produced by Harbin Meijun Pharmaceutical Co., Ltd. (test preparation, specification: omeprazole 20 mg+ sodium bicarbonate 1680 mg) and omeprazole sodium bicarbonate dry suspension produced by Santarus (Santarus) Company (reference preparation, trade name: ZEGERID) in Chinese volunteers. The bioequivalence of the two preparations was evaluated by the main pharmacokinetic parameters and relative bioavailability to provide a clinical basis for the drug registration application of the tested preparations. Besides, it is necessary to clarify how the metabolic genotypes of CYP2C19 influence this drug’s pharmacokinetics.

## Methods

### Compliance with ethics guidelines

This research was conducted under the guidance of the Declaration of Helsinki, Good Clinical Practice (GCP) guidelines of the China Food and Drug Administration (CFDA) and authorized by the independent ethics committee of Tongji Medical College, Huazhong University of Science and Technology (No. (2018)186-1). Written informed consent from each volunteer is required before any procedure can proceed. Clinical trial Registration Numbers: ChiCTR2200058964. The date of registration is 20/04/2022.

### Subjects

This study included 40 subjects. The subjects were 18–65 years (including 18 and 65 years old). The male’ s body weight was ≥ 50.0 kg, and for females ≥ 45.0 kg, the range of body mass index (BMI) was from 19 to 26 kg/m^2^. All of them were good at communicating with investigators and they could understand and follow the requirements during the whole experiment. The exclusion criteria were as follows: history of any chronic disease; the current or recent illness that could have influenced the pharmacokinetic (PK) parameters of this drug; smoking or alcohol addiction; use of prescription/over-the-counter drugs within 14 days before taking the study drug; pregnant women; lactating women; subjects with a history of allergy to other benzimidazoles. Informed consent was obtained from all participants.

### Study design

**Figure 1:**
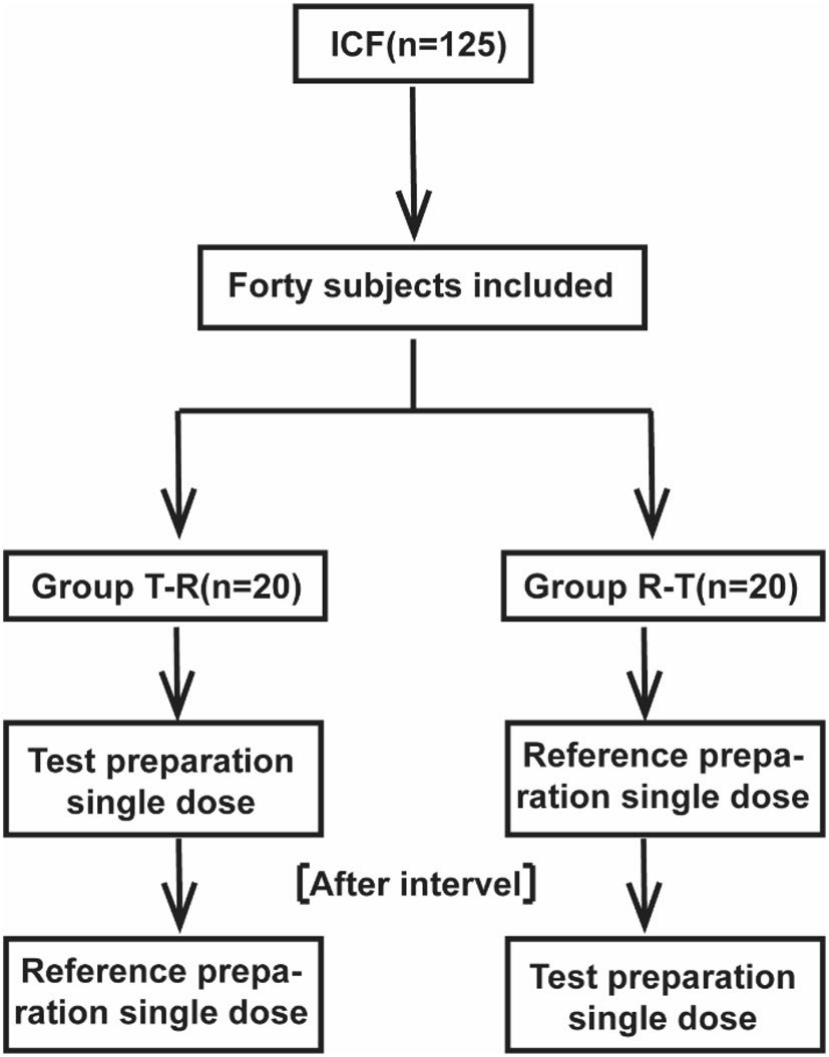
Trial profile.

This study was a single-dose and two-period PK study, shown in Fig. 1.

Forty subjects were randomly divided into two groups, with 20 patients in each group. The drugs were given to the patients of each group in the order of T-R and R-T in two cycles, with a single dose of 1 bag (each bag: 20 mg omeprazole + 1680 mg sodium bicarbonate). The subjects in each group fasted after 21:00 the night before administration. Collecting plasma samples at 0 h (within 60 min before administration) and after administration of 5 min, 10 min, 15 min, 20 min, 30 min, 45 min, 1 h, 1.5 h, 2 h, 3 h, 4 h, 6 h, 8 h, 10 h, 12 h (a total of 16 points) and stored at − 80 °C until analysis. The blood samples were centrifuged at 1700 g (4 °C) for 10 min within 1 h of collection. The centrifuged plasma was immediately aliquoted into 2 tubes with corresponding labels (1 for testing, 1 for backup, the volume of plasma in each tube should not be less than 500 µL) and stored at ATPase in the activated form 80 °C until analysis. The total time from whole blood collection to centrifugation to separate plasma and then stored in ATPase in the activated form 80 °C refrigerator should not exceed 2 h.

### Analytic methods

The concentration of omeprazole in EDTA-K2 anticoagulant human plasma was determined by HPLC–MS/MS, quantified by internal standard (internal standard: omeprazole-D3). The sample pretreatment method was the protein precipitation method. And the linear range of the omeprazole plasma concentration determination method was 4–4000 ng mL^−1^, the minimum quantitative limit was 4 ng mL^−1^.

### Pharmacokinetics analysis

The pharmacokinetic (PK) parameters evaluated in this study included maximum plasma omeprazole concentration (C_max_) and time to reach the maximum plasma concentration (T_max_) obtained directly from non-interpolated data, as well as the area under the plasma concentration curve of omeprazole at 0-t after administration (AUC_0–t_) using the linear trapezoidal method to calculate. Terminal elimination rate constant λz and the apparent terminal elimination half-life (T_1/2_) were also needed. AUC_0–∞_ (the AUC from time 0 to infinity) used the formula: AUC_0–∞_ = AUC_0–t_ + Ct/λ(T_1/2_ = 0.693/λz) to calculate.

### Safety evaluations

Safety was assessed by gathering electrocardiograms, vital signs, physical examinations, and clinical laboratory results. AEs were divided into mild, moderate, or severe to determine the relationship between the study drug and AEs according to the criteria declared by the World Health Organization.

### Statistical methods

SAS 9.4 software was used for statistical analysis. After logarithmic conversion, C_max_, AUC_0–t,_ and AUC_0–∞_ performed a two-way unilateral t-test to calculate the 90% confidence interval of the geometric mean ratio of omeprazole C_max_, AUC_0–t,_ and AUC_0–∞_ in the plasma of tested preparation T and reference preparation R. When the 90% confidence interval of the geometric mean ratio of C_max_, AUC_0–t,_ and AUC_0–∞_ between the tested and the reference preparation was within the equivalent interval of 80.00–125.00%, the bioequivalence of the two preparations could be determined. Besides, the nonparametric method was used to evaluate the T_max_ of test and reference preparation.

## Results

### Subjects

A total of forty volunteers (24 males and 16 females) were recruited. The mean age of this volunteer group was 24.24 ± 4.08 years. And the mean height and body weights were 165.46 ± 7.91 cm and 60.21 ± 7.97 kg, with a mean BMI of 21.87 ± 1.76 kg m^−2^.

### Safety and tolerability

Two formulations of Omeprazole and Sodium bicarbonate powder have safety, and healthy volunteers were well tolerated throughout the trial. There were no significant changes in all data or information of physical examination, vital signs, laboratory examination results, or 12 lead ECG compared with those before administration. In this study, 12 subjects had 19 adverse events; the incidence rate was 30%. Of the 12 subjects, 9 belong to the IM group, 2 are PM, and 1 is EM. Among them, there were 1 case of metabolic and nutritional diseases (1 case of hyperuricemia), 3 cases of infection and infection diseases (3 cases of upper respiratory tract infection), and 10 cases of various examinations (1 case of white blood cell count increased, 1 case of elevated alanine transfers, 1 case of urinary white blood cell positive, 2 cases of hemoglobin decrease, 2 cases of the abnormal electrocardiogram T wave, 1 case of urine red blood cell positive, 1 case of blood pressure drop, platelet count decrease 1 case), 5 cases of gastrointestinal diseases (3 cases of abdominal distension, 1 case of nausea, 1 case of gastroesophageal reflux disease)（Table S1）. Adverse events occurred in 11 cases in the T–R dosing sequence and 8 cases in the R–T dosing sequence. The severity of adverse events was mild in 11 cases and moderated in 1 case. Except for one subject with reduced hemoglobin who reported no discomfort and refused to come to the hospital for review, the other adverse events had improved or disappeared/relapsed after follow-up. Neither the reference preparation nor the test preparation had serious adverse reactions.

### Pharmacokinetic parameters

**Figure 2:**
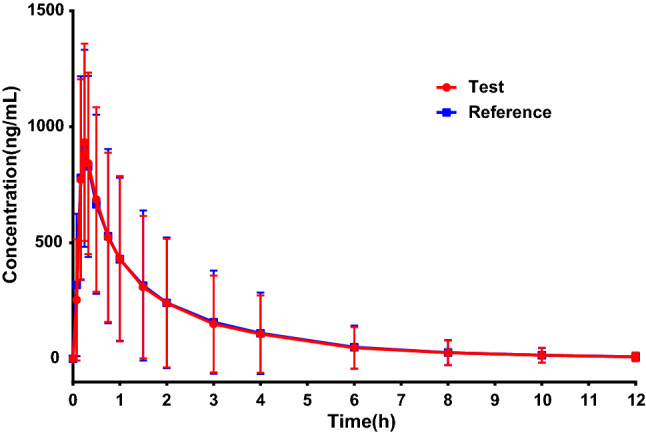
Plasma concentration–time profiles of Test preparation and Reference preparation.

The pharmacokinetic parameters and concentration–time profiles of the test and reference preparation were listed below (Table 1, Fig. 2). After a single fasting oral administration of test or reference preparation in 40 healthy subjects, the calculated AUC_0–t_ of test and reference were 1530.61 ± 1584.30 ng h mL^−1^ and 1553.81 ± 1618.30 ng h mL^−1^, AUC_0–∞_ were 1572.21 ± 1642.10 ng h mL^−1^ and 1594.10 ± 1676.30 ng h mL^−1^, the T_max_ was 0.25 h (0.17, 0.75 h) and 0.25 h (0.08, 1 h) and the C_max_ was 981.50 ± 431.72 ng mL^−1^ and 1010.35 ± 430.97 ng mL^−1^, respectively. And there were no statistically significant differences in the extent and rate of drug exposure between T and R preparation, *p* > 0.05.

**Table 1:** Summary of main pharmacokinetic parameters of two formulations of Omeprazole and sodium bicarbonate powder.

Parameter	Arithmetic mean ± SD (%CV) (N = 40)		*p*
	Test preparation	Reference preparation	
C_max_ (ng mL^−1^)	981.50 ± 431.72 (43.99)	1010.35 ± 430.97 (42.66)	0.77
T_max_ (h)	0.25 (0.17,0.75)	0.25 (0.08,1.00)	0.32
AUC_0–t_ (ng h mL^−1^)	1530.61 ± 1584.30 (103.51)	1553.81 ± 1618.30 (104.15)	0.95
AUC_0–∞_ (ng h mL^−1^)	1572.21 ± 1642.10 (104.45)	1594.10 ± 1676.30 (105.16)	0.95
T_1/2_ (h)	1.15 ± 0.71 (61.77)	1.15 ± 0.68 (59.05)	0.99
λz (h^−1^)	0.774 ± 0.308 (39.84)	0.759 ± 0.290 (38.19)	0.82
CL (L h kg^−1^)	27.96 ± 23.69 (84.75)	27.03 ± 20.19 (74.74)	0.85
Vd (L kg^−1^)	27.42 ± 13.38 (48.8)	25.02 ± 9.43 (37.69)	0.36

C_max_ maximum blood concentration, T_max_ time to maximum blood concentration, AUC_0–t_ Area under curve from time 0 (baseline) to time t, AUC_0–∞_ Area under curve from zero to infinity, T_1/2_ elimination half-life, λz apparent end elimination rate constant, CL plasma clearance, V_d_, apparent volume of distribution. Data was presented in mean ± standard deviation.

*p* > 0.05, No significant.

### Bioequivalence

As summarized in Table 2, comparing test and reference preparation, the GMR of C_max_, AUC_0–t,_ and AUC_0–∞_ were 95.20%, 97.47%, 97.68%, respectively. And the 90% CIs ranged from 88.48 to 102.43%, 94.04 to 101.02% and 94.27 to 101.21%, all of which were within 80.00–125.00%. Besides, the results showed no significant difference in T_max_ between test (0.28 h) and reference preparation (0.27 h), *p* > 0.05.

**Table 2:** Bioequivalence analysis of main pharmacokinetic parameters of subjects after single oral administration of test preparation T and reference preparation R.

Parameter	GM(N = 40)			%CV	90% CI	*p*
	T	R	GMR			
C_max_	876.69	920.89	95.20	19.61	88.48–102.43	0.99
AUC_0–t_	986.67	1012.31	97.47	9.52	94.04–101.02	1.00
AUC_0–∞_	1008.20	1032.19	97.68	9.44	94.27–101.21	1.00

C_max_ maximum blood concentration, AUC_0–t_ Area under curve from time 0 (baseline) to time t; AUC_0–∞_ Area under curve from zero to infinity, GM = geometric mean, GMR = geometric mean ratio, GMR values report the geometric mean ratio and 90% confidence interval. *p* > 0.05, not significant.

### Effects of CYP2C19 phenotypes on PKs

**Figure 3:**
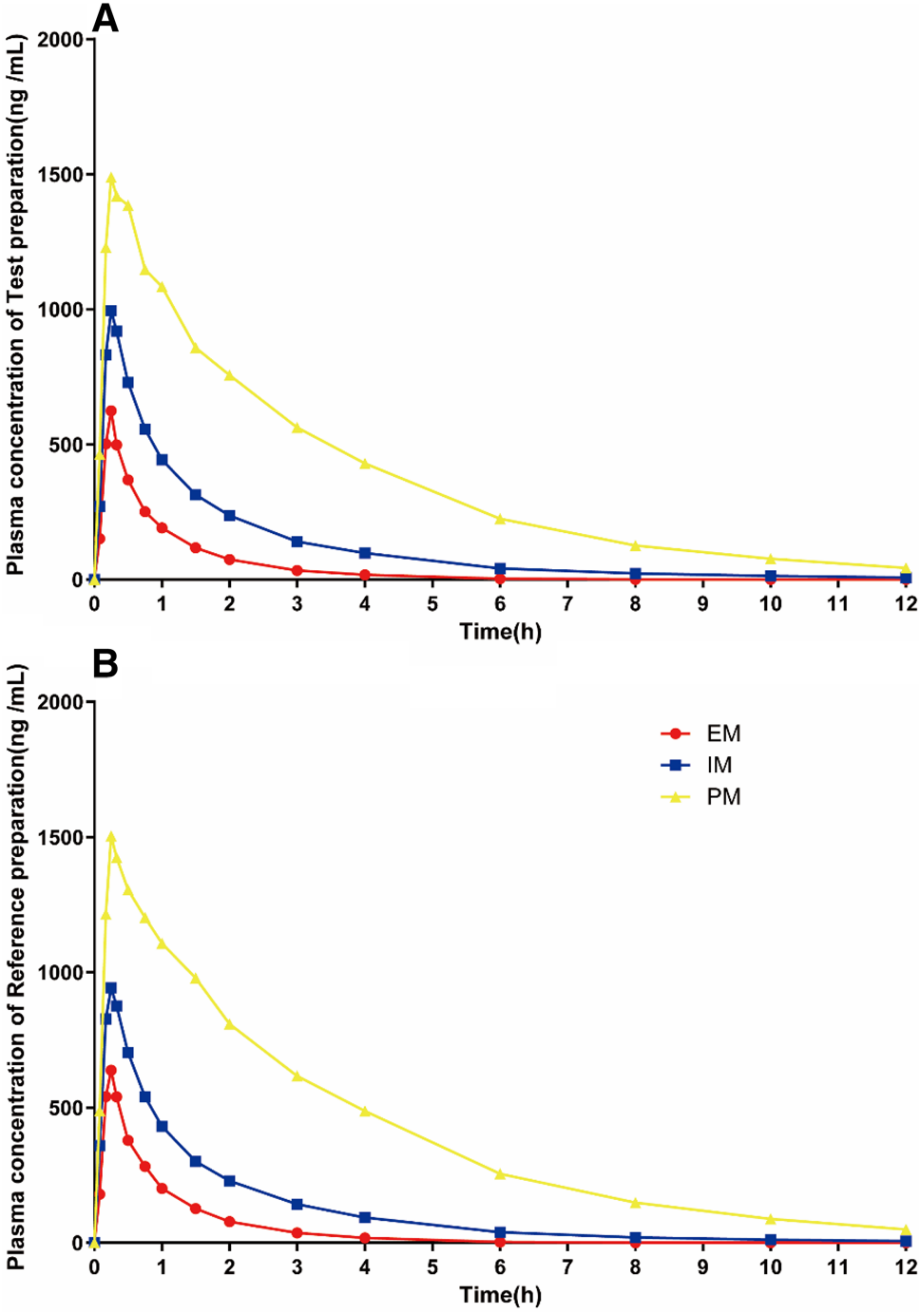
Plasma test (**A**) and reference, (**B**) preparation concentrations-time profiles in relation to CYP2C19 phenotypes. (PK = pharmacokinetic, PM = poor metabolizer, IM = intermediate metabolizer, EM = extensive metabolizer.).

**Figure 4:**
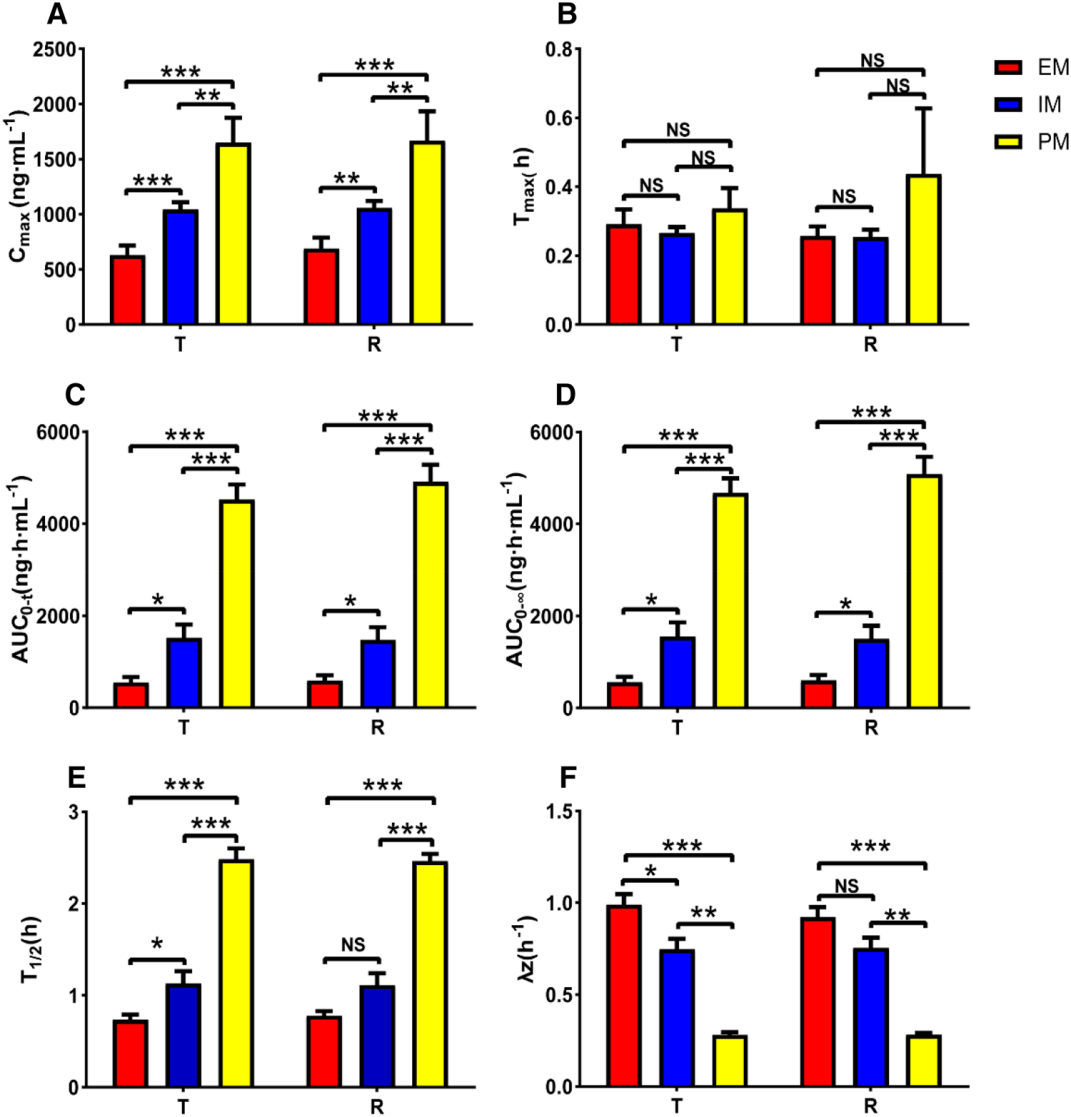
Comparison of omeprazole C_max_ (**A**), T_max_ (**B**), AUC_0–t_ (**C**), AUC_0–∞_ (**D**), T_1/2_ (**E**) and λz (**F**) between different CYP2C19 genotypes after oral administration of test preparation and reference preparation. *p* > 0.05, not significant, *p* < 0.05, *, *p* < 0.05, **, *p* < 0.001, ***.

Of the 40 subjects completing the study, 40 subjects were divided into EM (CYP*1/*1, N = 12), IM (CYP*1/*2, N = 23, CYP*1/*3, N = 1), and PM (CYP*2/*2, N = 4). The AUC_0–t_, AUC_0–∞_, C_max_, T_1/2_, λz, CL, and V_d_ of test and reference preparation in each metabolic genotype were shown as mean ± SD in Table 3. According to the CYP2C19 phenotype, the mean plasma concentration–time profiles of the test preparation and the reference preparation were shown in Fig. 3. The plasma concentration of test and reference preparation in PM were much higher than that in EM and IM. In the PM of the test preparation, the C_max_ was 1650.00 ± 451.66 ng mL^−1^, which was significantly higher than that in EM (633.25 ± 295.94 ng mL^−1^, *p* < 0.01) and IM (1044.21 ± 320.07 ng mL^−1^, *p* < 0.001). And in the PM, the AUC_0–t_ was 4526.56 ± 651.59 ng h mL^−1^, which was significantly higher compared with EM (552.90 ± 391.42 ng h mL^−1^, *p* < 0.001) and IM (1520.14 ± 1437.90 ng h mL^−1^, *p* < 0.001). Likewise, T_1/2_ was also the highest in PM compared with the other groups (*p* < 0.001). In contrast, the drug clearance of the PM was the lowest among the three groups (*p* < 0.001). However, in terms of absorption, there was no significant difference in the T_max_ in the three groups of the test preparation in vivo (*p* > 0.05). Similarly, significant differences were also found in C_max_, AUC_0–t_, AUC_0–∞,_ T_1/2_, and λz of the reference preparation between different CYP2C19 genotypes. In contrast, there were no differences in the T_max_ of reference in three groups, *p* > 0.05. In general, no matter whether it was the test or the reference preparation, the C_max_, AUC_0–t_, and AUC_0–∞_ in the EM, IM, and PM groups were gradually increased (*p* < 0.05), and T_1/2_ and λz were gradually decreased (*p* < 0.05), except that there was no difference in T_1/2_ and λz between the EM and IM groups in the reference preparation (Fig. 4). For that, the CYP2C19 phenotypes have little effect on the absorption of drugs in the human body, and the main effect lies in drug metabolism.

**Table 3:** The PK of test and reference preparation about CYP2C19 phenotypes.

CYP2C19 gene polymorphisms	AUC_0–t_ (ng h mL^−1^)		AUC_0–∞_		C_max_(ng mL^−1^)		T_1/2_ (h)		T_max_ (h)		λz (h^−1^)		CL (L h kg^−1^)		V_d_ (L kg^−1^)	
	T	R	T	R	T	R	T	R	T	R	T	R	T	R	T	R
EM (N = 12)	552.90 ± 391.42	591.09 ± 401.03	563.59 ± 378.58	600.82 ± 387.43	633.25 ± 295.94	690.50 ± 338.19	0.74 ± 0.19	0.78 ± 0.17	0.25 (0.17, 0.75)	0.25 (0.17, 0.5)	0.99 ± 0.2	0.92 ± 0.19	50.95 ± 28.09	45.23 ± 23.32	35.03 ± 18.45	24.68 ± 11.92
IM (N = 24)	1520.14 ± 1437.90	1475.31 ± 1356.51	1558.7 ± 1495.48	1508.26 ± 1402.83	1044.21 ± 320.07	1060.33 ± 303.17	1.13 ± 0.65	1.11 ± 0.63	0.25 (0.17, 0.5)	0.25 (0.08, 0.5)	0.75 ± 0.27	0.76 ± 0.26	20.4 ± 11.91	21.72 ± 11.78	25.55 ± 9.06	26.78 ± 7.98
PM (N = 4)	4526.56 ± 651.59	4913.02 ± 738.77	4679.13 ± 627.44	5089.02 ± 742.13	1650.00 ± 451.66	1670.00 ± 530.28	2.48 ± 0.24	2.46 ± 0.16	0.3 (0.25 0.5)	0.29 (0.17, 1)	0.28 ± 0.03	0.28 ± 0.18	4.32 ± 0.54	4.28 ± 0.25	15.79 ± 3.32	15.79 ± 0.74
*p* (EM/IM)	< 0.05	< 0.05	< 0.05	< 0.05	< 0.001	0.002	0.049	0.08	0.96	0.67	0.01	0.06	0.04	0.5	< 0.001	< 0.001
*p* (EM/PM)	< 0.001	< 0.001	< 0.001	< 0.001	< 0.001	< 0.001	< 0.001	< 0.001	0.48	0.24	< 0.001	< 0.001	0.06	0.15	0.005	0.004
*p* (IM/PM/)	< 0.001	< 0.001	< 0.001	< 0.001	0.002	0.002	< 0.001	< 0.001	0.41	0.14	0.002	0.001	0.045	0.009	0.01	0.007

C_max_ maximum blood concentration, T_max_ time to maximum blood concentration, AUC_0–t_ Area under curve from time 0 (baseline) to time t, AUC_0–∞_ Area under curve from zero to infinity, T_1/2_ elimination half-life, λz apparent end elimination rate constant, CL plasma clearance, V_d_, apparent volume of distribution. Data was presented in mean ± standard deviation. *p* > 0.05, not significant.

### Effects of gender on PK

As in the Table 4, for the test preparation, the C_max_ in females was 1030.38 ± 533.83 ng mL^−1^, higher than 948.92 ± 356.95 ng mL^−1^ in males. Although T1/2 in females was 1.05 ± 0.62 h, slightly lower than that in males 1.21 ± 0.72 h, (*p* = 0.46), the AUC_0–t_ and AUC_0–∞_ in females were similar to those in males (1531.43 ± 1800.01 and 1569.40 ± 1855.29 ng h mL^−1^ vs. 1530.06 ± 1463.91 and 1574.08 ± 1525.61 ng h mL^−1^, *p* = 0.99) (Table 4). And there was no difference in T_max_ between the females and males, *p* = 0.79. After a single dose of the reference, It seemed that the C_max_ in females was 1040.00 ± 501.05 ng mL^−1^, higher than in males 990.58 ± 387.56 ng mL^−1^, and because of the shorter T_1/2_ (1.05 ± 0.62 h in females vs. 1.21 ± 0.72 h in males), the AUC_0–t_ and AUC_0–∞_ tended to be lower in females than in males (1514.00 ± 1784.04 and 1549.42 ± 1834.64 ng h mL^−1^ in females vs. 1580.36 ± 1537.25 and 1623.89 ± 1602.28 ng h mL^−1^ in males), but all of these PK parameters between the females and males had no significant differences. Besides, there was no difference in T_max_ after the non-parametric test.

**Table 4:** The PK of test and reference preparation in males (N = 24) and females (N = 16) healthy volunteers.

Parameter	Test preparation			Reference preparation		
	Male	Female	*p*	Male	Female	*p*
C_max_ (ng mL^−1^)	948.92 ± 356.95	1030.38 ± 533.83	0.57	990.58 ± 387.56	1040.00 ± 501.05	0.73
T_max_ (h)	0.25 (0.17, 0.75)	0.25 (0.17, 0.5)	0.79	0.25 (0.17, 1)	0.25 (0.08, 0.5)	0.98
AUC_0–t_ (ng h mL^−1^)	1530.06 ± 1463.91	1531.43 ± 1800.01	0.99	1580.36 ± 1537.25	1514.00 ± 1784.04	0.90
AUC_0–∞_ (ng h mL^−1^)	1574.08 ± 1525.61	1569.40 ± 1855.29	0.99	1623.89 ± 1602.28	1549.42 ± 1834.64	0.89
T_1/2_ (h)	1.22 ± 0.75	1.03 ± 0.66	0.42	1.21 ± 0.72	1.05 ± 0.62	0.46
λz (h^−1^)	0.72 ± 0.29	0.85 ± 0.33	0.21	0.73 ± 0.3	0.8 ± 0.27	0.45

C_max_ maximum blood concentration, T_max_ time to maximum blood concentration, AUC_0–t_ AUC from time 0 (baseline) to time t, AUC_0–∞_ AUC from zero to infinity, T_1/2_ elimination half-life, λz apparent end elimination rate constant. Data was presented in mean ± standard deviation. *p* > 0.05, not significant.

## Discussion

Omeprazole has been widely recognized and used as the first generation of new acid inhibitors once discovered. Different enteric coatings are necessary to protect acid unstable PPI from gastric acid degradation within the stomach, which has the potential detriment of PPI absorption delayed^18^. But omeprazole sodium bicarbonate dry suspension can overcome this problem. Sodium bicarbonate can not only protect omeprazole from being destroyed by gastric acid^23^, but also can quickly neutralize gastric acid, increase the pH value in the stomach, relieve some clinical symptoms, and activate the proton pump channel in a large amount. Omeprazole can directly act on the proton pump channel to inhibit the secretion of gastric acid by the proton pump. The first purpose of this study is to find out the bioequivalence of the test and reference preparation. After the single administration, the exposure of the test and reference preparation was similar. The GMR (90%CI) for C_max_, AUC_0–t_, and AUC_0–∞_ were all between 80 and 125%. In addition, both preparations were well tolerated without any serious adverse events. There were no newly reported adverse events in the present study, and there was no significant difference in the frequency of drug-related adverse events between these two formulations.

Omeprazole has highly variable pharmacokinetics, of which CYP2C19 is a major influencing factor^24,25^. The CYP2C19 gene is extensively polymorphic with 39 known alleles^26^, belonging to an important drug-metabolizing enzyme in the liver cytochrome P450 enzyme series. The frequency alleles of CYP2C19 tend to differ in relation to race^27,28^. The CYP2C19*2 and CYP2C19*3 are responsible for PM alleles, mainly found in Asians. The CYP2C19*2 has an allele frequency of 25–30% in Asians and about 15% in whites^29^, and CYP2C19*3 has an allele frequency of about 2–7% in Asians^29^ while 0.04% in whites^30^. Of the 40 volunteers in the present study, the allele distributions for CYP2C19 were *1, *2, and *3 at 60%, 38.75%, and 1.25% close to the ratio in the Asian described above. Besides, it was similar to the allele distributions for CYP2C19 in other Chinese studies, that the *1, *2, and *3 were 58.2–69.7%, 24.7–37.7%, and 2–4.1%^31–34^. While the frequency of the CYP2C19 EM, IM, and PM genotypes in the present study were 30%, 60, and 10%, which is similar to other CYP2C19 gene polymorphisms performed in Chinese, the proportions of EM, IM, and PM were 27.5%, 57.5%, and 15%, respectively^35^. The results in the present study showed that CYP2C19 was crucial for omeprazole pharmacokinetics in vivo. The CL was significantly higher in EM, compared with it in PM and IM. The most representative PK parameters-AUC, reflecting the drug clearance, which was significantly increased in PM 8.2, 3 times in EM and IM, consistent with previous studies^29^. The other PK parameters C_max_ and T_1/2_ were also significantly different according to different genotypes (Fig. 4). Alterations in PK parameters of omeprazole were found in several studies in different races^36–40^. In the EM group of the present study, the AUC_0–t_ of omeprazole was 552.9 ± 391.42 ng h mL^−1^, which was higher than 250.5 ± 16.1 ng h mL^−1^ in West Asian^37^, but not different from the reports in Whites (635.5 ± 259.7 ng h mL^−1^)^36^, and East Asians (618.3 ± 141.9 ng h mL^−1^ in Japanese^38^ and 713.49 ± 555.56 ng h mL^−1^ in Korean^39^). This result showed that Caucasian has the least growth of omeprazole AUC_0–t_ between the EM group and PM group than those in East and West Asians. In the EM group, the omeprazole AUC_0–t_ of Caucasian was comparable those of East Asians, but still higher than that of West Asians. In the PM group, the omeprazole AUC_0–t_ Caucasian was significantly lower than that of East Asians, but still higher than that of West Asians. Our data is closer to the reported pharmacokinetic data in Chinese^40^ and Japanese population. In PM group, the omeprazole AUC_0–t_ value is significantly higher than that of Caucasians and West Asians. At the same time, the increment of omeprazole AUC_0–t_ from EM to PM groups is close to 8 times, which is much higher than 5 times enhance in those of Caucasians and Koreans, and lower than 9 times in West Asians. Omeprazole total exposure differs among different races for the same metabolic phenotype, specifically in the PM. There in the PM, the C_max_ was 635.5 ± 259.7 ng mL^−1^ in Caucasians and 538.6 ± 33.5 ng mL^−1^ in East Asians, lower than 1070.2 ± 185.3 ng mL^−1^ in Japanese and 1650.00 ± 451.66 ng mL^−1^ in present study. In the EM group, the C_max_ of this study was 633.25 ± 295.94 ng mL^−1^, higher than that 285 ± 82.9 ng mL^−1^ in Caucasians and 251.1 ± 46.2 ng mL^−1^ in Japanese, and 152.4 ± 9.94 ng mL^−1^ in West Asians was lowest. These showed that the absorption of omeprazole in EM and PM of this study were higher than those in Caucasian, West Asians and Japanese. In the EM, the C_max_ in the Caucasians was similar with that of Japanese, but higher than that in West Asians. But in the PM, the C_max_ in the Caucasians was close to that in West Asians, and lower than that in Japanese. Besides, the data showed that the increment of omeprazole C_max_ from EM to PM in this study is close 3 times, which is lower than 4 times enhance in those of West Asians and Japanese, and higher than 2 times in Caucasians. For the half-time of the omeprazole in these populations, in the EM group, the T_1/2_ in this study was 0.74 ± 0.19 h, close to 0.71 ± 0.1 h in Caucasians, but lower than 0.9 ± 0.01 h in West Asians and 1.09 ± 0.08 h in Japanese. And in the PM, the T_1/2_ was 2.48 ± 0.24 h, close to 2.68 ± 0.3 h in Caucasians, 2.42 ± 0.18 h in West Asians and 2.41 ± 0.15 h in Japanese, which indicated that the half-time of omeprazole in the PM of these populations are similar.

In addition, gender may also affect the pharmacokinetic parameters of drugs by affecting the activity of CYP2C19, thus changing the drug’s efficacy. Shabnam Nazir's study showed that the C_max_ and AUC_0–t_ of omeprazole in females (2.913 ± 0.61 μg mL^−1^ and 8.74 ± 2.23 μg h mL^−1^) was significantly higher than those (2.006 ± 0.98 μg mL^−1^ and 6.67 ± 4.32 μg h mL^−1^) in males. At the same time, the C_max_ and C_max_ of 5-hydroxyomeprazole and omeprazole siphon of women were greatly higher than those of men, which meant that there were significant differences in CYP2C19 activity between females and males in Pakistani^41^. In another study in the Korean population, for the same CYP2C19 genotype, Korean women metabolized omeprazole faster than Korean men^42^. However, An Iranian study reported that there was no difference in the hydroxylation index of omeprazole between females and males^43^. And this study showed that the PK parameters of the test and reference preparation had no differences between males and females, which was consistent with the other studies in Chinese^44^ and Whites^45^. Taken together, the sex dependence of CYP2C19 activity may be related to the race included in the study.

There are several limitations in this study. First, only healthy volunteers were enrolled in the present study, the omeprazole concentration–time profile of which may differ from the acid-related disordered patients. Secondly, the number of enrolled volunteers should be expanded to meet the needs of volunteers for pharmacokinetic research in PM. Thirdly, the efficacy of omeprazole was not evaluated, which exerted its drug effect by inhibiting gastric acid secretion. This study failed to evaluate the intragastric 24 h pH or serum gastrin for its efficacy.

## Conclusion

The results showed that the GMR (90% CI) Cmax, AUC_0–t_, and AUC_0–∞_ were all between 80 and 125%, which meant that the test and reference preparation are bioequivalent. And there were no serious AEs that occurred during the trials, indicating that both medications are well tolerated and have exemplary safety in healthy Chinese volunteers. Besides, our study demonstrated that CYP2C19 gene polymorphism significantly differed in the PK parameters of omeprazole sodium bicarbonate dry suspension.

## Supplementary Information

Supplementary Information.

## Data Availability

The datasets used and analyzed during the current study available from the corresponding author on reasonable request.
